# Adenocarcinoma of Mullerian origin: review of pathogenesis, molecular biology, and emerging treatment paradigms

**DOI:** 10.1186/s40661-015-0008-z

**Published:** 2015-05-12

**Authors:** Lauren Patterson Cobb, Stephanie Gaillard, Yihong Wang, Ie-Ming Shih, Angeles Alvarez Secord

**Affiliations:** Division of Gynecologic Oncology, Department of Obstetrics and Gynecology, Duke Cancer Institute, Duke University Medical Center, Durham, NC 27710 USA; Division of Medical Oncology, Department of Internal Medicine, Duke University Medical Center, Durham, NC 27710 USA; Department of Gynecology and Obstetrics, Johns Hopkins University School of Medicine, Baltimore, MD 21205 USA

**Keywords:** Adenocarcinoma, Mullerian origin, Epithelial ovarian carcinoma, Fallopian tube carcinoma, Peritoneal carcinoma

## Abstract

Traditionally, epithelial ovarian, tubal, and peritoneal cancers have been viewed as separate entities with disparate origins, pathogenesis, clinical features, and outcomes. Additionally, previous classification systems for ovarian cancer have proposed two primary histologic groups that encompass the standard histologic subtypes. Recent data suggest that these groupings no longer accurately reflect our knowledge surrounding these cancers. In this review, we propose that epithelial ovarian, tubal, and peritoneal carcinomas represent a spectrum of disease that originates in the Mullerian compartment. We will discuss the incidence, classification, origin, molecular determinants, and pathologic analysis of these cancers that support the conclusion they should be collectively referred to as adenocarcinomas of Mullerian origin. As our understanding of the molecular and pathologic profiling of adenocarcinomas of Mullerian origin advances, we anticipate treatment paradigms will shift towards genomic driven therapeutic interventions.

## Introduction

Adenocarcinoma of Mullerian origin was first described by Dr. Swerdlow in 1959 [1]. The original manuscript entitled, “Mesothelioma of the pelvic peritoneum resembling papillary cystadenocarcinoma of the ovary,” described a patient with a malignant left-sided pelvic mass. The mass surrounded the left fallopian tube without mucosal involvement; bilateral ovaries and the right tube were negative for disease. Histologically, the tumor closely resembled a papillary ovarian cystadenocarcinoma. Dr. Swerdlow theorized that while ovarian or tubal carcinoma was unlikely, the tumor probably developed from tissue with a similar embryological origin as the ovary (specifically, the pelvic peritoneum, fallopian tubes, or uterus). He ultimately concluded that the cancer arose from the pelvic peritoneum [1]. In retrospect, this case represents the earliest documentation of adenocarcinoma of Mullerian origin. There is a growing body of evidence that suggests this terminology applies to epithelial ovarian, peritoneal, and tubal cancers, as well as select cancers previously designated as “cancers of unknown primary” (CUP). Select endometrial cancers may also be included in future classifications, but as the treatment paradigms are different, we chose not to include them in this review.

Recent data regarding the genetics and histopathology of epithelial ovarian cancer (EOC) has improved our understanding of ovarian carcinogenesis. These results and current hypotheses indicate that epithelial ovarian, peritoneal, and tubal cancers are not distinct entities but represent a spectrum of disease that originates in the Mullerian compartment. Due to this new information, the FIGO staging classification for ovarian, tubal, and peritoneal cancers was revised (Table 1) [2]. Tubal and peritoneal cancers are now included in the ovarian cancer staging classification, and the primary site designated when possible [2,3]. This new staging exemplifies our current understanding of the relationship between these disease entities and challenges our previous classification of ovarian, peritoneal, and tubal cancers. We and others assert that this group of gynecologic cancers should be collectively designated as adenocarcinomas of Mullerian origin. In this review, we will focus on the incidence, classification, and origin of Mullerian adenocarcinomas. We will also review the molecular and pathologic profiling that support the concept of adenocarcinomas of Mullerian origin as a unified entity and will assist in diagnostic and treatment paradigms.

**Table 1:** **Ovarian cancer staging (FIGO 2013 vs. FIGO 1988)**

**FIGO (1988)**	**FIGO (2013)**
I: Tumor limited to the ovaries	I: Tumor confined to ovaries or fallopian tube(s)^a^
IA: Tumor limited to 1 ovary (capsule intact), no tumor on ovarian surface, no malignant cells in ascites or peritoneal washings	IA: Tumor limited to 1 ovary (capsule intact) or fallopian tube; no tumor on ovarian or fallopian tube surface; no malignant cells in the ascites or peritoneal washings
IB: Tumor limited to both ovaries (capsules intact), no tumor on ovarian surface, no malignant cells in ascites or peritoneal washings	IB: Tumor limited to both ovaries (capsules intact) or fallopian tubes; no tumor on ovarian or fallopian tube surface; no malignant cells in the ascites or peritoneal washings
IC: Tumor limited to 1 or both ovaries with any of the following: capsule ruptured, tumor on ovarian surface, malignant cells in ascites or peritoneal washings	IC: Tumor limited to 1 or both ovaries or fallopian tube(s) with any of the following:
	IC1: Surgical spill intraoperatively
	IC2: Capsule ruptured before surgery or tumor on ovarian or fallopian tube surface
	IC3: Malignant cells in the ascites or peritoneal washings
II: Tumor involves 1 or both ovaries with pelvic extension	II: Tumor involves 1 or both ovaries or fallopian tubes with pelvic extension (below pelvic brim) or primary peritoneal cancer^b^
IIA: Extension and/or implants on uterus and/or tube(s); no malignant cells in ascites or peritoneal washings	IIA: Extension and/or implants on uterus and/or fallopian tubes and/or ovaries
IIB: Extension to other pelvic tissues; no malignant cells in ascites or peritoneal washings	IIB: Extension to other pelvic intra-peritoneal tissues
IIC: Pelvic extension (IIA or IIB) with malignant cells in ascites or peritoneal washings	
III: Tumor involves 1 or both ovaries with microscopically confirmed peritoneal metastases outside the pelvis and/or regional lymph node metastasis	III: Tumor involves 1 or both ovaries or fallopian tubes, or primary peritoneal cancer, with cytologically or histologically confirmed spread to the peritoneum outside the pelvis and/or metastasis to the retroperitoneal lymph nodes
IIIA: Microscopic peritoneal metastasis beyond pelvis	IIIA1: Positive retroperitoneal lymph nodes only (cytologically or histologically proven)
	IIIA1(i): Metastasis up to 10 mm in greatest dimension
	IIIA1(ii): Metastasis more than 10 mm in greatest dimension
	IIIA2: Microscopic extra-pelvic (above the pelvic brim) peritoneal involvement with or without positive retroperitoneal lymph nodes
IIIB: Macroscopic peritoneal metastasis beyond pelvis, 2 cm or less in greatest dimension	IIIB: Macroscopic peritoneal metastasis beyond the pelvis up to 2 cm in greatest dimension, with or without metastasis to the retro-peritoneal lymph nodes (includes extension of tumor to capsule of liver and spleen without parenchymal involvement of either organ)
IIIC: Peritoneal metastasis beyond pelvis more than 2 cm in greatest dimension and/or regional lymph node metastasis	IIIC: Macroscopic peritoneal metastasis beyond the pelvis more than 2 cm in greatest dimension, with or without metastasis to the retro-peritoneal lymph nodes (includes extension of tumor to capsule of liver and spleen without parenchymal involvement of either organ)
IV: Distant metastasis (excludes peritoneal metastasis)	IV: Distant metastasis excluding peritoneal metastases
	IVA: Pleural effusion with positive cytology
	IVB: Parenchymal metastases and metastases to extra-abdominal organs (including inguinal lymph nodes and lymph nodes outside of the abdominal cavity)^c^

^a^It is not possible to have stage I peritoneal cancer.

^b^Dense adhesions with histologically proven tumor cells justify upgrading apparent stage I tumors to stage II.

^c^Extra-abdominal metastases include transmural bowel infiltration and umbilical deposits.

Adapted from Zeppernick F, Meinhold-Heerlein I. The new FIGO staging system for ovarian, fallopian tube, and primary peritoneal cancer. *Archives of gynecology and obstetrics.* Aug 1 2014.

## Review

### Incidence

It is difficult to discern how many annual deaths occur due to adenocarcinomas of Mullerian origin. While EOC caused approximately 14,030 deaths in the United States in 2013 [4] and 151,905 deaths worldwide in 2012 [5], it is unclear exactly how many deaths were caused by peritoneal and tubal cancers. Peritoneal and tubal carcinomas have been considered rare malignancies and separate entities from ovarian carcinomas; thus, epidemiologic studies have proven difficult [6]. Tubal carcinomas account for only 0.14-1.8% of gynecologic malignancies [7,8]. In the United States, from 1995–2004, the age adjusted incidence rates for tubal and peritoneal carcinomas were 3.7 and 6.8 per million, respectively [6]. Newer theories indicate that the number of peritoneal and tubal cancers may be grossly underestimated.

Additionally, CUP accounts for 3-5% of malignant epithelial cancers [9] and in 2012, there were an estimated 31,000 new cases of CUP in the United States [10]. Potentially 5% of CUP may originate in the female reproductive system based on data from post mortem autopsy studies [9,11]. It is important to recognize the adenocarcinoma of Mullerian origin subset of CUP when it occurs, because these cancers will typically have a more favorable prognosis and sensitivity to platinum-based chemotherapeutic regimens [12]. Identification of adenocarcinoma of Mullerian origin, specifically in patients with CUP, will guide appropriate treatment options, and provide information regarding prognosis [9,12].

### Current classification

#### Epithelial ovarian cancer classification

EOC classification has changed significantly over the past decade. The most recent proposed division of EOC includes two distinct histologic groups: type I and type II cancers. It should be noted that the type I and type II classification is generally used to broadly classify ovarian neoplasms for research purposes based on their unique clinical and molecular genetic features [13]. The classification was not meant to be used for clinical purposes. Type I tumors include low-grade serous and low-grade endometrioid cancers, as well as mucinous, clear cell, and transitional cell carcinomas. Tumors in this category typically develop from atypical proliferative borderline tumors, benign cystic lesions, or endometriosis. Transitional cell tumors and mucinous tumors do not typically have Mullerian features, but may develop from cortical inclusion cysts and Walthard cell nests [14]. However, there is an uncommon subtype of mucinous tumors which does demonstrate Mullerian (endocervical) characteristics [15,16]. Generally, type I tumors are more indolent, present at an earlier stage, are confined to the ovary, and are often large. When type I tumors, specifically clear cell and mucinous cancers, are not detected early, they usually have a worse prognosis than type II cancers [14].

Type II cancers account for approximately 75% of EOC and the vast majority of ovarian cancer deaths. These include high-grade serous and high-grade endometrioid carcinomas, as well as carcinosarcomas and undifferentiated carcinomas. These cancers are typically aggressive and diagnosed at a later stage [13,14,17]. Until recently the origin or precursor lesion for the type II cancers was unknown [18]. However, it is now recognized that the precursor lesion exists in the fallopian tube, as discussed later in this review [14,17,19-21].

#### Fallopian tube cancer classification

As mentioned above, per the 2014 FIGO staging classification, tubal and peritoneal cancers are now considered collectively with ovarian cancer [2]. Regarding histologic classification, serous tubal carcinomas are most frequent (49.5-83.3%), followed by endometrioid (8.3%-50%), mixed (3.9-16.7%), transitional (11.7%), undifferentiated (7.8-11.3%), mucinous (3%-7.6%), and clear cell (1.9%) cancers [7]. These histologic subtypes are similar to the proportions seen in EOC; however, clear cell histology is more common in EOC, while transitional cell and undifferentiated histology is more frequent in tubal cancers [7,8]. In the past, the diagnosis of tubal carcinoma was made based on pathologic criteria with at least one the following: 1) the primary tumor arises from the endosalpinx in the fallopian tube 2) the histologic pattern resembles epithelial mucosa and is often papillary in nature 3) there is a clear transition between benign and malignant epithelium if the wall is involved, and 4) there is no evidence of malignancy in the ovaries or endometrium, or if tumor is present, there is less tumor than is present in the fallopian tube [7].

#### Peritoneal cancer classification

Peritoneal carcinomas have been called multiple names including peritoneal papillary serous carcinoma, peritoneal mesothelioma, primary peritoneal carcinoma, and normal-sized ovary carcinoma syndrome. In 1993, the Gynecologic Oncology Group established specific guidelines for the diagnosis of peritoneal carcinoma: 1) ovaries are of normal size or enlarged only as a result of a benign process 2) extraovarian involvement is greater than surface ovarian involvement 3) ovarian involvement does not show evidence of cortical invasion, is confined to the ovarian surface epithelium and cortical stroma and is less than 5×5 mm, and 4) histologically, the cancer is primarily of serous type, appearing similar or identical to ovarian serous adenocarcinoma of any grade [22]. Historically, peritoneal cancers have been reported to be more frequently multifocal with diffuse micronodular spread and more difficult to cytoreduce compared to EOC [23]. In 1994, Fowler et al. characterized the natural history of peritoneal adenocarcinoma of Mullerian origin. He reported that most were classified as serous histology and had either omental disease or diffuse carcinomatosis [12]. Currently, while viewed as separate entities, patients with peritoneal carcinoma are commonly included in ovarian cancer trials, treated similarly to ovarian cancer with cytoreductive surgery and platinum-based chemotherapy [24], and now considered collectively with ovarian and tubal cancer in the staging guidelines [2].

### Theories regarding adenocarcinoma of Mullerian origin

**Figure 1:**
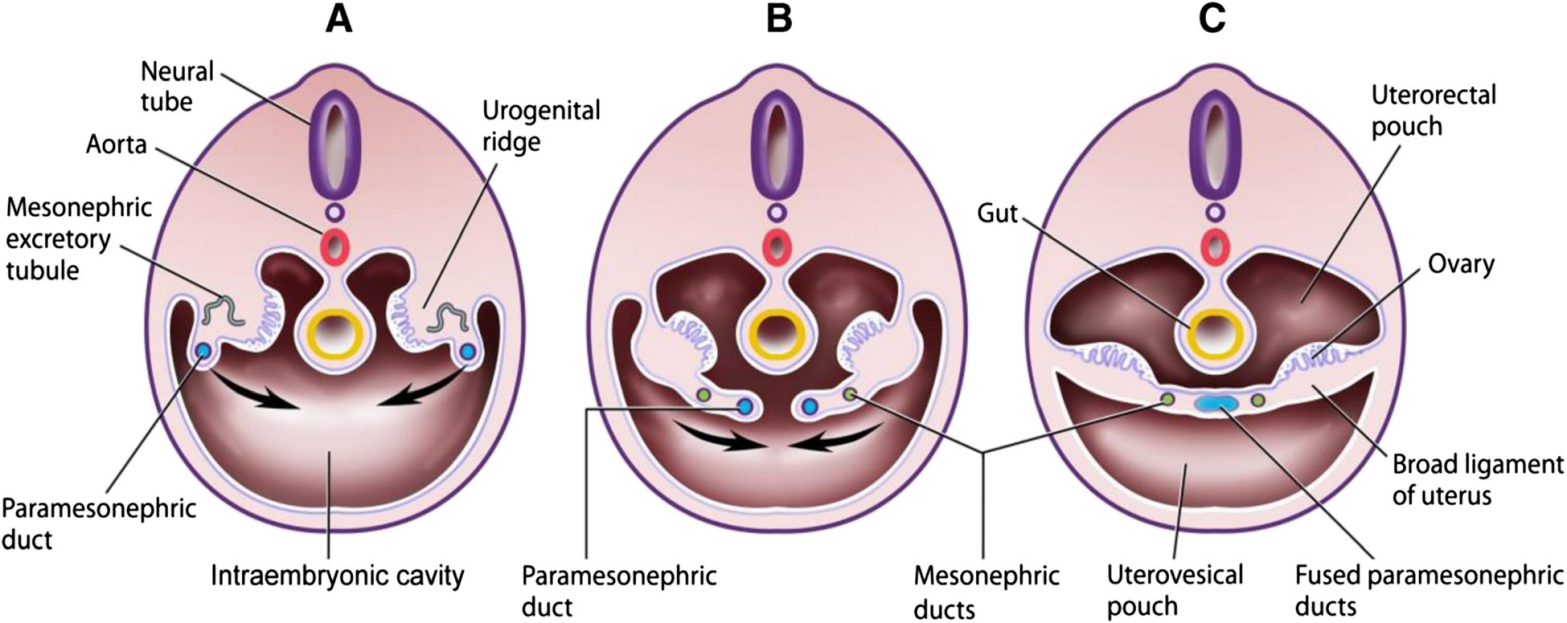
Transverse section through the urogenital ridge. Progressing from **A** to **C**, the paramesonephric ducts approach each other through the intraembryonic cavity and fuse in the midline forming the Mullerian structures (broad ligament of the uterus, fallopian tubes and uterus). *Adapted from Swadler, T.W. Langman’s Medical Embryology, 10^th^ Edition. Philadelphia: Lippincott Williams & Wilkins; 2006: 246.

Comprehension of the embryologic origin of the Mullerian system is critical to understanding the theories surrounding the origin of ovarian, peritoneal, and tubal cancers. Ovarian surface epithelium (OSE) is derived from the coelomic epithelium in early development. The coelomic epithelium is derived from the mesoderm, consists of the epithelial lining of the intraembryonic body cavity or coelom, and overlies the intraembryonic body cavity (which will become the peritoneum), including the area that will develop into the gonadal structures. During fetal development, near the area that will form the gonadal structures, the coelomic epithelium invaginates to give rise to the Mullerian (paramesonephric) ducts (which will ultimately differentiate to become the fallopian tubes, uterus, cervix, and upper vagina). Therefore, while the reproductive organs and peritoneum originate from distinct pathways, the Mullerian epithelia, OSE, and peritoneal (coelomic) epithelium have a close developmental relationship (Figure 1) [25].

Ovarian carcinogenesis was previously thought to occur through the invagination of the OSE into the underlying stroma to form inclusion cysts. Metaplasia of the epithelium on the wall of these cysts was proposed to transform the OSE into the aforementioned cell types and their corresponding tumors: serous, mucinous, clear cell, endometrioid and transitional cell carcinomas. This theory seems unlikely for two reasons: (1) the normal ovary does not bear resemblance to the morphologic phenotype of any of these tumors, and (2) it suggests that ovarian cancers develop de novo. However, cancers typically develop in a stepwise fashion from a benign lesion to a malignancy [14]. An alternate theory proposed that ovarian tumors develop from nearby paraovarian and paratubal cysts consisting of Mullerian-type epithelium, called the “secondary Mullerian system.” As the tumors grow from these cysts, they infringe upon the ovary, compress it, and eventually obliterate it, making it appear as though it is ovarian in origin [14,26]. This theory seems unlikely as well, given that paratubal and paraovarian cysts rarely contain precursor lesions resembling serous, clear cell, or endometrioid carcinomas [14]. However, the secondary Mullerian system may also include endosalpingiosis, endometriosis, and endocerviocosis. Metaplasia from these tissues are commonly observed in ovarian malignancies [27]; thus, this theory may account for the development of some ovarian cancers [27]. The most recent theory proposes that the majority of serous, endometrioid, and clear cell “primary ovarian” cancers actually develop from the fallopian tube and endometrium, the “primary Mullerian system” and will be discussed further in this review [14,27].

#### Origin of type I EOC

In type I EOC, there is considerable evidence that clear cell and endometrioid carcinomas may originate from endometriosis. The pathogenesis of endometriosis is complex and theories include retrograde menstruation as well as metaplasia of extrauterine cells. Retrograde menstruation would indicate that endometrioid and clear cell cancers develop from endometrial tissue, the primary Mullerian system, which secondarily involves the ovary [14]. Several studies have demonstrated an increased risk of ovarian cancer in the setting of endometriosis [28-30]. A meta-analysis of endometriosis in EOC concluded that the prevalence of endometriosis was significantly higher in women with clear cell cancers (35.9%) and endometrioid carcinomas (19%), compared to those with serous (4.5%), and mucinous (1.4%) cancers [31].

The origin of mucinous carcinoma is unclear. It is commonly accepted that a majority of mucinous cancers involving the reproductive tract are actually metastases from extraovarian sites, usually gastrointestinal in origin. True primary ovarian mucinous carcinomas are uncommon, accounting for only 3% of ovarian carcinomas, although one recent theory includes mucinous metaplasia of Brenner (transitional cell) tumors [32]. Brenner tumors and mucinous carcinomas (intestinal type) may share similar histogenesis at the tubal peritoneal junction from transitional cell nests that exist there [13]. As mentioned previously, an uncommon subtype of mucinous tumors does demonstrate Mullerian (endocervical) characteristics [15,16]. Most advanced mucinous cancers are likely metastatic gastrointestinal and pancreaticobilliary cancers that involve the ovary and peritoneum.

**Figure 2:**
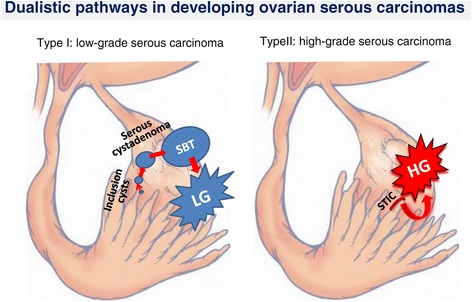
The dualistic pathways in developing low-grade and high-grade “ovarian” serous carcinoma. The Type I pathway develops from the presumed fallopian tube epithelial stem cells that disseminated into the ovulation site where those stem cells form surface inclusion cysts. Those cysts may continue to grow into serous cystadenomas and clonally develop into serous borderline tumors, which represent the precursor lesions of low-grade serous carcinomas. In contrast to the step-wise tumor progression pathway as observed in Type I serous tumors, in the Type 2 pathway, many high-grade serous carcinomas arise as a result of dissemination of their precursor lesions, serous tubal intraepithelial carcinomas (STICs), in the fallopian tube fimbriated ends.

With regard to low-grade serous carcinoma (LGSC), multiple studies support the step-wise progression of serous cystadenoma or adenofibroma to atypical proliferative serous tumor (atypical serous borderline tumor), to noninvasive micropapillary serous borderline tumor, to invasive LGSC (Figure 2) [33]. Previously, we reported identical hallmark *KRAS* mutations in serous borderline ovarian tumors and their associated Mullerian inclusion cysts, suggesting a relationship between the two. It is unclear if Mullerian inclusion cysts represent a precursor lesion, signify metastatic disease from the primary borderline tumor, or develop due to a metaplastic field effect [34]. While *KRAS* and *BRAF* mutations are common in borderline tumors, *NRAS* mutations are only seen in carcinomas and may represent the requisite oncogenic switch to invasive serous cancer [35]. There is also evidence to support the development of LGSC from fallopian tube precursors or papillary tubal hyperplasia [14,27,36,37].

#### Origin of type II EOC

**Figure 3:**
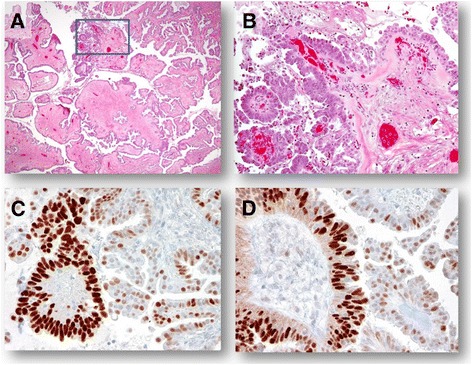
A high-grade serous carcinoma arises from a serous borderline tumor. **A**. A low-magnification view shows a focal high-grade serous carcinoma developing from the papillae (square) in a background of a typical serous borderline tumor. **B**. A higher magnification demonstrates enlarged and atypical high-grade serous carcinoma cells that organize in a papillary architecture. **C** and **D**. Immunohistochemistry of p53 shows that high-grade serous carcinoma cells are diffusely positive for p53, a pattern consistent with a missense *TP53* mutation while the adjacent epithelial cells from the background serous borderline tumor are only focally and weakly positive, a pattern consistent with a wild-type *TP53* sequence.

An observation by Piek et al. would eventually revolutionize hypotheses regarding the origin of high-grade serous carcinoma (HGSC). In 2001, Piek and colleagues examined specimens from women who had undergone a risk reducing bilateral salpingo-oophorectomy who were either BRCA mutation carriers or had a strong family history of ovarian cancer. Fifty percent of the specimens had preinvasive dysplastic lesions (later coined “serous tubal intraepithelial carcinoma” (STIC)) that resembled HGSC. Almost all specimens had high levels of p53 protein accumulation (indicating accumulation of a nonfunctional p53 protein due to a *TP53* genetic mutation). Initially this new information was interpreted to mean that tubal carcinoma should be included in the in the spectrum of BRCA-associated disease [38]. In 2003, Piek et al. reevaluated their findings and hypothesized that lesions in the fallopian tube epithelium are the precursor lesions for hereditary and *BRCA*-mutated ovarian cancer [19]. Further studies performed in *BRCA* mutation carriers revealed that benign areas of the tubal epithelium overexpressing p53 nonfunctional protein may represent a precursor to STIC in the pathway to the development of HGSC [21,39]. STICs are present in the majority of serous ovarian (59-67%), peritoneal (67%), and tubal (100%) carcinomas [17,21,25]. In contrast, STICs were not identified in mucinous, endometrioid, or carcinosarcoma histologic subtypes [40]. In addition, further studies have reported identical *TP53* mutations in paired STIC and the concurrent HGSC indicating a clonal relationship between them [20]. While most HGSCs arise from STICs, alternative pathways in developing HGSC also exist. For instance, a small number of HGSCs appear to arise from serous borderline tumors or LGSCs (Figure 3) [27,41].

While it is not clear how STIC is related to the development of peritoneal cancers, some have hypothesized that sloughed tubal cancer cells disseminate into the peritoneal cavity and implant accordingly. While Sood et al. proposed hematogenous spread of ovarian cancer cells with a predilection for implantation in the omentum [42], perhaps both modes of metastasis (peritoneal and hematogenous dissemination) play a role in Mullerian carcinogenesis.

Overall, contemporary data indicate that endometrioid and clear cell cancers arise from endometrial tissue with the fallopian tube as a conduit between the uterus, ovary, and peritoneum; serous cancers from STICs in the fallopian tube [36]; Brenner and mucinous cancers from transitional-type epithelium found at the tubal-peritoneal junction that secondarily implant or metastasize to the ovary and peritoneal surfaces; and rare mucinous cancers from endocervical mucinous neoplasms. Therefore, while historically documented as separate processes, we would argue that ovarian, tubal, and peritoneal cancers should be uniformly referred to as adenocarcinomas of Mullerian origin given their similar pathogenesis.

### Disease outcomes for adenocarcinoma of Mullerian origin

In a recent meta-analysis, Sørensen et al. compared serous peritoneal, tubal and ovarian cancer with regards to risk factors, epidemiology, clinicopathology, and molecular biology to address whether these diseases should be considered separately. When comparing peritoneal cancers with ovarian cancers, even though most of these studies were limited by small sample sizes, nine studies showed no significant difference in survival [43-51]. Only three studies showed poorer survival for peritoneal cancers [52-54]; however, two of these studies had a small number of patients with peritoneal cancer [52,53]. When comparing tubal cancers to ovarian cancers, Sørensen et al. sited three studies showing similar survival between these two disease entities [54-56] and two showing improved survival for tubal cancers [57,58]. The studies by Usach et al. [57] and Wethington et al. [58] were large studies using the SEER database and did not include information on residual disease after debulking surgery. All of the aforementioned studies had limitations. Most of these studies included small sample sizes, utilized differing definitions of optimal cytoreduction, and failed to include detailed information regarding pathology, surgery, treatment regimens, recurrences, and confounding risk factors, making them difficult to compare and then generalize their findings. Despite an extensive literature search by Sørensen and colleagues, the small number of studies as well as their limitations preclude definitive conclusions regarding survival outcomes between ovarian, tubal, and peritoneal cancers.

### Biomarkers and pathologic assessment for adenocarcinoma of Mullerian origin

Serum biomarkers are useful for the detection, response assessment, and prognosis in a variety of solid tumors, including adenocarcinomas of Mullerian origin. Cancer antigen 125 (CA125) is the only biomarker commonly used for monitoring treatment response and cancer progression in EOC [59], as well as tubal and peritoneal cancers [60]. CA125 is a glycoprotein encoded by the gene MUC16. In patients with advanced EOC, CA125 is elevated (greater than 35 u/mL) approximately 90% of the time. However, in patients with early stage EOC, CA125 is elevated only 50-60% of the time. CA125 is an excellent marker for ovarian cancer, but is nonspecific and can be abnormal in other benign and malignant indications. CA125 expression levels also vary by histology and are elevated in 85% of serous, 65% of endometrioid, 40% of clear cell, and 36% of undifferentiated adenocarcinomas [59].

There are additional markers that are useful to distinguish between various solid tumors. These include carbohydrate antigen 19–9 (CA19-9), carcinoembryonic antigen (CEA), and human epididymis protein 4 (HE4). CA19-9 is member of the Lewis blood group antigens and is elevated in 27% and 76% of serous and mucinous ovarian cancers, respectively. CEA is a glycoprotein that is expressed in 25-50% of women with EOC and over 80% of patients with colorectal carcinomas. Human epididymis protein 4 (HE4) is overexpressed in serous and endometrioid carcinomas. Unlike CA125, HE4 is more specific to ovarian malignancy and serum levels are usually not elevated with nonmalignant processes [59]. A subset analysis of premenopausal patients enrolled in a prospective clinical trial (NCT00315692) demonstrated that HE4 had a sensitivity of 88.9% and a specificity of 91.8% for the detection of malignancy. In this analysis, invasive malignancy was ruled out for 98% of premenopausal women with an elevated CA-125 and a normal HE4 level [61]. There are other additional markers that have been used in combination with CA125, including cancer antigen 15–3 (CA15-3) and tumor associated glycoprotein 72 (TAG-72). Although CA15-3 is elevated in 57-71% of ovarian malignancies (versus 2-6% of benign ovarian processes), it has a low specificity for ovarian cancer and is primarily used for the diagnosis of breast malignancies. TAG-72 is expressed more commonly in gastrointestinal and pancreatic tumors as well as mucinous ovarian carcinomas [59]. Biomarkers can be useful for identifying adenocarcinomas of Mullerian origin in women with CUP, as well as following response to treatment.

### Pathological analysis of adenocarcinoma of Mullerian origin

Ovarian, tubal, and peritoneal cancers have similar pathologic findings which vary based on histologic subtype, but not by primary site of origin. We describe common histopathologic and immunophenotype findings for adenocarcinomas of Mullerian origin stratified by the various subtypes. Pathologic findings support a clear link between serous ovarian, tubal, and peritoneal cancers. However, information regarding pathologic similarities between tubal and peritoneal clear cell, mucinous, and endometrioid carcinomas is minimal given the relatively rare frequency of these histologic subtypes.

#### High-grade serous carcinoma

##### Histopathology

HGSCs of the ovary, fallopian tube, and peritoneum are almost identical in histopathology. Microscopically, the architecture could vary from glandular to complex papillary to solid pattern, with the tumor cells infiltrating or replacing the surrounding normal tissues. The papillae are usually large, irregularly branching, and highly cellular. Psammoma bodies may be present in varying numbers, but are rarely as numerous as in LGSC. The marked cytologic atypia and frequent mitotic figures (including atypical ones) characterize HGSC. The tumor cells are enlarged, with high nuclear/cytoplasmic ratio and great variation in size. Tumor giant cells are commonly seen. The nuclei are of high-grade with vesicular chromatin and prominent nucleoli [33].

##### Immunophenotype

**Figure 4:**
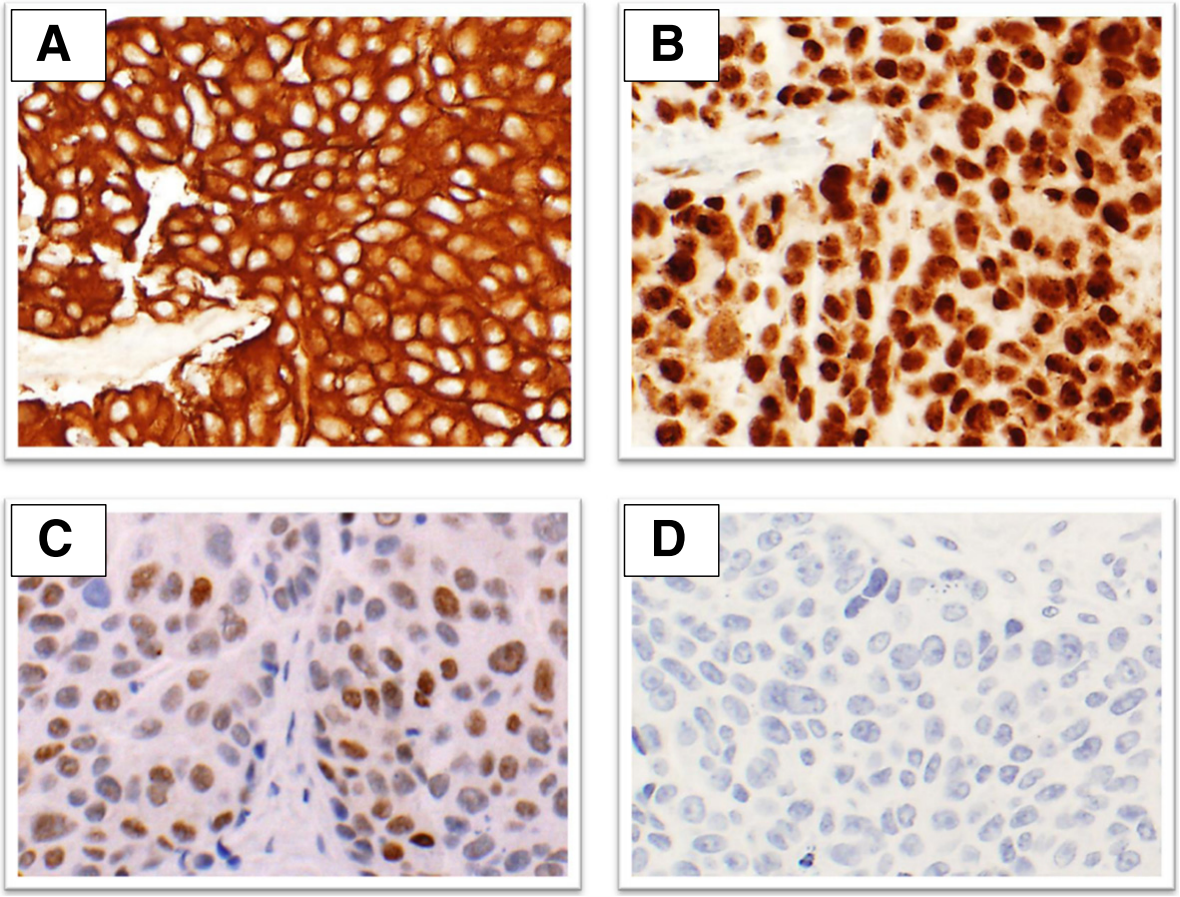
Representative microscopic sections of high-grade serous adenocarcinoma of Mullerian origin demonstrating positive immunostaining for **(A)** CK-7, **(B)** WT-1, **(C)** PAX-8, and **(D)** negative immunostaining for CK-20.

Immunophenotypically, ovarian and tubal HGSCs strongly and diffusely express p16, and CK7; express WT-1, PAX-8, estrogen receptor, CA125 and E-cadherin in most cases; do not express Her-2, calretinin, or CK20; and have a high Ki67 proliferative index (Figure 4) [33,62-65]. The staining pattern for p53 protein is usually consistent with either a missense mutation (diffusely and intensely positive) or nonsense/deletion type mutation (completely negative) [33]. Overall, peritoneal serous carcinomas almost always demonstrate the same immunohistochemistry pattern as ovarian and tubal HGSCs, with minor and inconsistent differences in WT-1, b-catenin, vimentin and CK20 expression [62,66-69].

#### Low-grade serous carcinoma

##### Histopathology

Similar to HGSC, there is strong evidence to support the tubal origin of LGSC [36]. In general, LGSC is characterized by micropapillae and small round nests of neoplastic cells that infiltrate the stroma in a haphazard pattern, with infrequent mitoses and only mild variation in tumor cell size and shape of nuclei. The nuclear/cytoplasmic ratio may be high but the nuclei are uniform, small, and round to oval. Psammoma bodies are common and may be numerous. Necrosis or multinucleated tumor giant cells are not features of LGSC. In contrast to HGSC, LGSC is usually associated with a non-invasive serous borderline component [33].

##### Immunophenotype

As previously discussed, the precursor lesions for LGSC are presumed to be epithelial inclusion cysts (leading to serous cystadenoma/adenofibroma, to atypical serous borderline tumor, to noninvasive micropapillary serous borderline tumor, to invasive LGSCs). These epithelial inclusion cysts were previously thought to arise from invaginations of the OSE that undergo metaplasia; however, the inclusion cysts may originate from tubal epithelia that secondarily implant on disrupted OSE and invaginate [36]. Li et al. demonstrated that OSE primarily has a mesothelial phenotype (calretinin(+)/PAX8(−)), while the majority of epithelial inclusion cysts demonstrate a tubal phenotype (calretinin(−)/PAX8(+)) [37]. It is not surprising then that LGSCs also express PAX8. Additionally, they express ER and WT-1, similar to HGSC. In contrast to HGSC, LGSC is characterized by decreased expression of p53 and p16 (usually negative, scattered, or patchy), and a lower Ki67 proliferative index [33].

Low-grade serous peritoneal carcinoma is a rare entity; and therefore, available information about this disease is minimal. Schmeler et al. were the first to clinically describe low-grade serous peritoneal cancer. Patients were confirmed to have low-grade serous carcinomas with destructive invasion. Microscopically the cancers had relatively uniform round to oval nuclei, mild to moderate atypia, evenly distributed chromatin, and no more than 12 mitoses per 10 high-power fields (HPF). Additionally, these patients met the previously described GOG criteria for peritoneal carcinoma. Specific immunostaining was not described [70].

#### Mucinous carcinoma

##### Histopathology

The majority of primary mucinous tumors of the ovary mimic features of gastric or pancreaticobilliary mucinous neoplasms, while another much less common subtype harbor Mullerian (endocervical) characteristics. A spectrum of morphologic changes from cystadenoma to atypical proliferative mucinous tumor (mucinous borderline tumor) to invasive mucinous carcinoma can often be appreciated. They are usually large unilateral neoplasms with a smooth capsule and confined to the ovary at diagnosis (stage I). Stromal invasion may be infiltrative or expansile [15,16]. Mucinous tumors of the fallopian tube and peritoneum are rare, but have been reported [7,8,24,71].

##### Immunophenotype

Ovarian mucinous carcinomas display predominance of CK7 over CK20. PAX-8 staining is much less frequent (40%) despite that it is almost universally positive (95–100%) in ovarian serous, endometrioid, and clear cell carcinomas [72]. WT1, ER, PR and p16 are not expressed in primary mucinous carcinomas. p53 protein may be present in 30% of cases, but strong and diffuse overexpression (as found in HGSC) is not characteristic [33,73-75].

#### Clear cell carcinoma

##### Histopathology

Clear cell carcinoma has also been associated with endometriosis and displays the following architectural and cytological features: papillary, tubulocystic or solid architecture; hobnail tumor cells with clear cytoplasm; and large, atypical nuclei with conspicuous nucleoli and only moderate polymorphism. Clear cell carcinoma papillae are distinguishable from those of serous carcinoma in that they are short and round, may show eosinophilic and hyalinized stroma, and are generally lined with only one or two layers of cells. Hyaline bodies are present in approximately 25% of cases. Mitoses are less frequent than in other types of ovarian carcinomas (usually < 5 per 10 HPF) [15,16]. While most literature focuses on “ovarian” clear cell carcinoma, there are published case reports of peritoneal and tubal clear cell cancers [76-79]. The histopathologic findings are similar to ovarian clear cell carcinomas, but immunostaining is not consistently available [76-78].

##### Immunophenotype

Generally, clear cell carcinomas display a CK7(+)/CK20(-) phenotype; express PAX-8; and lack expression of ER and WT-1. p53 and p16 are usually negative, weak, focal or patchy. Hepatocyte nuclear factor-1β (HNF-1β) is a specific and sensitive marker for ovarian clear cell carcinomas that is not expressed in HGSC [80-82].

#### Endometrioid carcinoma

##### Histopathology

Endometrioid carcinomas of the ovary highly resemble endometrioid carcinomas of the uterus in morphology. These cancers may coexist with endometriosis and arise from endometriotic cysts. They are mostly low-grade adenocarcinomas demonstrating a confluent glandular growth pattern with stromal disappearance, or evidence of stromal invasion and squamous metaplasia to varied degrees. Fifteen to thirty percent of patients have concurrent endometrial hyperplasia or carcinoma [83]. Similar histopathologic descriptions have been detailed in few case reports and case series of endometrioid carcinoma of the fallopian tube, but immunostaining was not described in detail [76,84-87]. The even rarer entity of endometrioid carcinoma of the peritoneum has been described in reference to extraovarian endometriosis-associated malignancy [88-90], however specific immunostaining has not been described.

##### Immunophenotype

Endometrioid carcinomas typically demonstrate CK7(+)/CK20(-) phenotype; express ER, PR and PAX-8; but lack WT-1 and p16 expression, as well as p53 overexpression. Exceptions to these patterns have been reported in poorly differentiated varieties, which overlap with HGSC in morphology [33,91].

### Molecular determinants of adenocarcinoma of Mullerian origin

The data regarding the molecular determinants of adenocarcinoma of Mullerian origin is primarily based on genomic studies of EOC [92]. However, some studies do include tubal and peritoneal cancers. Tothill et al. reported that serous and endometrioid cancers demonstrate a high degree of molecular heterogeneity and could be categorized into six subgroups based on gene expression profiling. Importantly, the primary site of disease could not be used as a classification parameter [93]. Tothill and colleagues reported six distinct subtypes referred to as C1-C6. C3 primarily consisted of serous low malignant potential tumors, while C6 primarily consisted of low-grade, early stage endometrial cancers. C1, C2, C4, and C5 mainly contained high-grade serous and high-grade endometrial cancers. Notably, C5 demonstrated a mesenchymal profile which was associated with relatively poor overall survival [93]. This finding is consistent with our understanding of cells acquiring the mesenchymal phenotype as they acquire invasiveness in the process of epithelial to mesenchymal transition (EMT). However, in the TCGA data set, a correlation between the mesenchymal subtype and survival was not seen [92]. Further evaluation is needed to confirm the associations between gene expression classifications and clinical outcome.

In 2013, Yang and colleagues took an integrated approach (as opposed to the previous transcriptome approach) to analyze serous cancers in the TCGA database and categorize the transcriptional subtypes into integrated mesenchymal and integrated epithelial subtypes. This new approach integrated mRNA expression with associated alterations in genomic, epigenetic, and miRNA systems. With this approach, Yang et al. were able to uncover a master miRNA regulatory network that consistently associated the integrated mesenchymal subtype of serous cancer with poor overall survival [94].

Additionally, intense interest has focused on using microarray data to identify molecularly-defined subgroups of women with HGSC who may benefit from anti-angiogenic therapy with bevacizumab. Gourley et al. evaluated a cohort of HGSC samples from the ICON7 study and identified three major subgroups; two with upregulation of angiogenic gene expression and one with upregulation of immune genes (and concurrent downregulation of angiogenic genes). Women in the immune subgroup had improved overall and progression-free survival (PFS) over the other two angiogenic subgroups. However, with the incorporation of bevacizumab, the immune subgroup had worse PFS (Hazard ratio (HR) = 1.73 (1.12-2.68)) and overall survival (HR, 2.00 (1.11-3.61)) compared to those treated with chemotherapy alone. In contrast, the pro-angiogenic subgroup treated with bevacizumab had a trend toward improved PFS [95]. Winterhoff and colleagues examined another subgroup of the ICON7 trial and reported that the greatest benefit from bevacizumab appeared in patients with serous carcinomas with the mesenchymal subtype (median PFS increased 9.5 months (25.5 [95%CI 21.1, NA] vs. 16 [95%CI 10.5, NA] months, p = 0.053)) [96]. The results from these studies suggest that bevacizumab therapy may be directed based on molecular subtypes. However, further assessment in a phase III integral biomarker trial is needed to determine if tumor-derived molecular classifications can direct individualized treatment with bevacizumab.

These studies all suggest possible new directions for therapies in serous ovarian cancer and may ultimately redefine our concept of ovarian cancer subtypes in an integrated molecular manner. While integral and integrated molecular biomarkers are critical to our understanding of cancer and new therapeutic strategies, our discussion of the molecular determinants of adenocarcinomas of Mullerian origin will be based on the dualistic Type I and II ovarian cancer model, recognizing that this model represents a simplistic categorization. We will also focus primarily on molecular findings in epithelial ovarian cancer, as detailed molecular data for tubal and peritoneal carcinomas is unavailable (Table 2).

**Table 2:** **Subtypes of adenocarcinomas of Mullerian origin**

	**High-grade serous**	**Low-grade serous**	**Mucinous**	**Clear cell**	**Endometrioid**
**Precursor lesion**	Tubal intraepithelial carcinoma	Atypical serous borderline tumor	Metaplasia of transitional cells or metastasis from GI primary tumor*	Atypical Endometriosis	Atypical Endometriosis
**Histologic features**	Positive: p16, CK7, WT-1, PAX-8, ER, CA125, E-cadherin (in most cases), p53	Positive: PAX8, ER, WT-1,	Positive: CK7, PAX-8 (40%), p53 (30%)	Positive: CK7, PAX-8, HNF-1β	Positive: CK7, ER, PR, PAX-8
	Negative: Her-2, calretinin, CK20	Negative: p53 and p16 (negative, scattered or patchy)	Negative: WT-1, ER, PR and p16	Negative: CK20, ER, WT-1, p53 and p16 (negative, weak, focal or patchy)	Negative: WT-1, p16, CK 20, p53
**Molecular aberrations**	*TP53* mutations	*BRAF, KRAS, NRAS* mutations	*KRAS* mutation	*PTEN* loss	*PTEN* loss
	*BRCA1/2* mutations		*HER2* amplification	*PIK3CA* mutation	*PIK3CA* mutation
	Chromosomal instability			*ARID1A* mutation	*ARID1A* mutation
**Risk factors**	Inherited *BRCA1/2* mutation			Endometriosis	Endometriosis
					Lynch syndrome

*Origin of Mullerian mucinous tumors is not definitively known.

#### High-grade serous ovarian, tubal, and peritoneal carcinoma

High-grade Mullerian cancers display predominantly serous histology, but also include some endometrial carcinomas, carcinosarcomas, and undifferentiated cancers. While less is known about the molecular profile of undifferentiated Mullerian tumors and carcinosarcomas, it appears that gene expression profiles and genetic alterations are very similar to those found in serous carcinomas [97,98]. These tumors exhibit a high level of genetic instability and are characterized by extensive chromosomal alterations and mutation of the tumor suppressor gene, *TP53* [99,100]. Mutation of *TP53* is an early event in the pathogenesis of HGSC and is found in STICs [20,101]. The presence of *TP53* mutations is nearly ubiquitous (>95%) in HGSC, thus it is not a useful prognostic or predictive biomarker [100].

The Cancer Genome Atlas (TCGA) Project recently analyzed mRNA and microRNA expression, exome sequencing of entire coding regions, copy number alterations, and methylation of 489 HGSC [92]. The high degree of genomic instability in these cancers is notable with 30 regional chromosomal aberrations (8 recurrent gains and 22 losses), 63 focal areas of amplification, and 50 focal deletions. By comparison, there were few mutations in individual genes identified. *TP53* was mutated in nearly all cases (>95%) and the next most commonly mutated genes were *BRCA1* and *BRCA2* (germline mutations present in 9% and 8% respectively, with somatic mutations in an additional 3% of cases). BRCA inactivation leads to defective repair of double stranded DNA breaks by homologous recombination. Although germline and somatic mutations of *BRCA1* and *BRCA2* account for <15% of cases, it was estimated that defects in homologous recombination genes, such as *EMSY*, *PTEN*, *RAD51C*, *ATM*/*ATR*, and Fanconi anemia genes, are present in 50% of all HGSC [92]. This may indicate that a large proportion of HGSC may be sensitive to treatments targeting DNA repair, such as PARP1 inhibitors.

Significant alterations have also been identified in the PI3K-AKT pathway. However, unlike type I cancers (such as clear cell or low-grade endometrioid cancers, which exhibit mutations in *PTEN* and *PIK3CA*), the pathway alterations in HGSCs are characterized by deletions (*PTEN*) and amplifications (*PIK3CA, KRAS,* and *AKT1/2*). Mutations in each individual gene account for <1% of the alterations. Similarly the retinoblastoma (Rb) signaling pathway is altered in 67% of cases with frequent down-regulation of *CDKN2A* (30%), deletion of *RB1* (8%), and amplification of *CCNE1* (20%), with few mutations found in these genes [92,102]. These data further support the finding that HGSC are characterized by generalized genomic instability rather than point mutations of driver genes.

Molecular signatures have been identified that are prognostic and/or predictive of response to therapy [103,104]. Whether or how these molecular signatures could guide clinical care is unclear. Confirmation of the initial results as well as biomarker-directed therapeutic trials are needed to determine if molecular signatures can be used to guide therapy in women with HGSC.

#### Low-grade serous carcinoma

Unlike HGSC, LGSCs do not exhibit chromosomal instability and are not associated with *TP53* or *BRCA* mutations [105,106]. Instead, mutations in the MAP kinase pathway are common with mutations in *BRAF* (38%) and *KRAS* (19%) the most frequent [107-109] as well as NRAS mutations [35]. These mutations also appear to be mutually exclusive [35,107]. In addition to the MAPK pathway mutations, LGSCs are more likely to exhibit increased expression of ER/PR, E-cadherin, PAX2, and IGF-1 compared to HGSC [110]. LGSC typically responds poorly to cytotoxic chemotherapy with an average response rate of only 4% in women with recurrent disease [111]. Based on studies suggesting that mutations in MAPK pathway genes act as driver mutations, inhibitors of the MAPK pathway, and in particular MEK inhibitors, are of great interest. Indeed, this has led to the trial of MAPK inhibitors for the treatment of women with recurrent LGSC. In a phase II trial of selumetinib, a MEK1/2 inhibitor, 15% of patients had an objective response to therapy and 65% had stable disease [112]. Further trials are ongoing, but these results present the potential of targeted individualized therapy based on a molecular understanding of the disease.

#### Mucinous carcinoma

Unlike HGSCs, in which *TP53* and *BRCA* mutations are most common, these mutations are relatively rare in mucinous tumors. Instead, the majority of mucinous tumors exhibit either *HER2* amplification or *KRAS* mutation [113]. The *KRAS* gene encodes the K-Ras protein, a key member of the RAS/RAF/MEK/ERK/MAP kinase signaling pathway that transduces various growth signals from the cell surface to the nucleus. *KRAS* mutations resulting in constitutive activation of the G protein are commonly found in codons 12, 13, and 61 and have been identified in a number of solid tumors [114]. *KRAS* mutations have been described in up to 68% of cases of mucinous ovarian cancer, while present in only 5% of non-mucinous tumors [113,115,116]. The large majority of mutations were identified in codon 12 (94%) [117]. *KRAS* mutations are thought to occur early in the development of these cancers as they are found in benign, low malignant potential, and borderline tumors of mucinous histology [117,118]. The high level of *KRAS* mutations in mucinous ovarian cancer may have treatment implications as targeted agents are being developed to target *KRAS* mutated tumors.

Overexpression/amplification of *HER2 (ERBB2)*, a member of the epidermal growth factor receptor family that acts upstream of *KRAS*, has been identified in up to 35% of mucinous ovarian cancer cases [113,119-121]. Ethnic differences may exist as *HER2* positivity was higher in Asian cohorts [119-121]. While no association was identified between *HER2* status and outcomes, responses of *HER2* amplified mucinous ovarian tumors to HER2 directed therapy have been reported [120-123].

#### Clear cell carcinoma

Similar to the other type I Mullerian carcinomas, clear cell carcinomas are not associated with chromosomal instability or mutations in *TP53* or *BRCA*. Notably, clear cell carcinomas of Mullerian origin exhibit distinctive gene expression profiles from other Mullerian histologies, while sharing significant expression patterns with clear cell tumors of the kidney and endometrium [124,125]. Ovarian clear cell carcinomas show increased activation of angiogenic, hypoxic cell growth, and glucose metabolic pathways and demonstrate increased sensitivity to anti-angiogenic therapies [126]. Clinical trials using anti-angiogenic tyrosine kinase inhibitors are currently in progress.

Nearly 50% of clear cell ovarian carcinomas were found to harbor *ARID1A* mutations resulting in loss of its encoded protein, BAF250a, a subunit of the SWI-SNF chromatin remodeling complex [127]. Loss of BAF250a expression is thought to be an early event in the pathogenesis of clear cell tumors as endometriotic cyst epithelium in direct contact with the tumor also exhibited loss of expression while cyst epithelium remote to the tumor did not [128]. Studies have shown that ARID1A acts as a tumor suppressor and coordinates with p53 protein to regulate cellular growth [129]. However, inactivating mutations of *ARID1A* alone do not appear to be sufficient for tumor formation, but likely require additional genetic alterations resulting in activation of the PI3K-Akt pathway [130,131]. Activating mutations in *PIK3CA* are found in 33% of cases, *PTEN* loss in 12%, with alterations in PI3K-Akt pathway occurring in 62% [131,132]. Preclinical studies suggest that targeting the PI3K-Akt pathway inhibits clear cell carcinoma tumor growth in a mouse model and that loss of ARID1A further sensitizes cells to PI3K- and Akt-inhibition [133,134]. Clinical trials of agents targeting the PI3K-Akt pathway are ongoing (NCT02142803, NCT01196429).

#### Endometrioid carcinoma

Similar to the dualistic pathway of pathogenesis of serous carcinomas, molecular profiling of high-grade endometrioid carcinomas are notable for mutations in *TP53* with the absence of other molecular alterations, while low-grade endometrioid carcinomas were strongly associated with microsatellite instability (20%), *CTNNB1* mutations (~50%), and *KRAS* mutations (up to 35%) [135-137]. High-grade endometrioid carcinomas were found to have a gene expression profile similar to HGSC [93]. Low-grade endometrioid carcinomas, however, are similar to clear cell adenocarcinomas in their association with endometriosis, expression of *ARID1A* mutations, and activation of the PI3K-Akt pathway. Ovarian endometrioid carcinomas are characterized by frequent somatic *ARID1A* inactivating mutations (30-55% of cases) [127,137,138]. Mutations typically are deletion or nonsense mutations which result in loss of protein expression [139]. ARID1A loss is associated with loss of PTEN and mutations in *PIK3CA* resulting in increased activation of the PI3K-Akt pathway [140]. It has been demonstrated in a genetically engineered mouse model that co-deletion of *ARID1A* and *PTEN* results in the formation of ovarian carcinoma with morphological and molecular features resembling human ovarian endometrioid carcinoma [130]. Activating mutations of *PIK3CA* are found in 20% of endometrioid carcinomas, while mutations in *PTEN* are present in 14-20%, and loss of heterozygosity of *PTEN* was present in 42% [136,137,141,142]. Loss of ARID1A has also been identified in endometrial hyperplasia with atypia, the precursor lesion of endometrioid carcinoma, and appears to be an early event in its pathogenesis [143]. However, despite the similarities between clear cell carcinomas and endometrioid carcinomas in *ARID1A* and *PI3K-Akt* pathway aberrations, protein array analysis showed differential expression between the two subtypes with endometrioid carcinomas expressing higher levels of steroid hormone receptors (ER and PR), and clear cell carcinomas expressing higher levels of Cyclin E, SMAD3, and e-cadherin [140]. Similarly, *BRAF* mutations were identified in 24% of endometrioid carcinomas, but were not identified in any case of clear cell carcinoma [107].

Other mutations frequently found in low-grade endometrioid carcinomas include mutations in *CTNNB1* (the gene that encodes beta-catenin) and mutations in mismatch repair genes. Mutations in *CTNNB1* are found in up to 50% of endometrioid ovarian tumors and are associated with improved outcomes [135-137]. Mutations typically result in over-expression of nuclear beta-catenin and increased transcription of down-stream target genes, such as the proto-oncogene *MYC*. These changes are present in a majority of borderline endometrioid ovarian tumors suggesting it is an early event in tumorigenesis [144]. Patients with Lynch syndrome are also at risk for developing EOC, most commonly the endometrioid subtype. Microsatellite instability has been detected in up to 20% of endometrioid tumors [136]. Similar to other Lynch-associated tumors, these tumors often exhibit abnormal mismatch repair protein expression with complete loss of MLH1, MSH2, MSH6, and/or PMS2 [145].

## Conclusions

Our review of the molecular, genetic, and histopathologic data supports the comprehensive inclusion of epithelial ovarian, tubal, and peritoneal cancers, as well as select CUP, as adenocarcinomas of Mullerian origin. While the dualistic Type I and II model of epithelial ovarian cancer suggests two main categories, it is unclear if this model can be extended to adenocarcinomas of Mullerian origin. However, it is clear that the different histologic subtypes within these categories are distinct with regard to clinical outcome, pathophysiologic, and molecular features which may have therapeutic implications. In light of the aforementioned advancements in genomics we propose a new nomenclature for this set of diseases. The terminology may include adenocarcinoma of Mullerian origin, followed by presumed primary site (ovary, fallopian tube, peritoneum), histologic subtype, and mutation status (if relevant). This type of nomenclature would appropriately capture the similarities among adenocarcinomas of Mullerian origin in both origin and histology, but recognize the unique molecular differences between them, all of which inform treatment decisions and prognosis. An example of such a classification could be “adenocarcinoma of Mullerian origin, fallopian tube primary, high-grade serous histology, *BRCA1* mutation." Currently, the standard treatment of adenocarcinomas of Mullerian origin includes cytoreductive surgery and multi-agent platinum-based chemotherapy. The advances made in understanding the underlying molecular determinants of adenocarcinomas of Mullerian origin, as well as development of targeted therapeutics, will enable the implementation of genomic-driven treatment decisions in the future, elucidation of novel targets that can be used in preventive strategies, and better identification of precursor lesions that will yield improved survival outcomes.
